# Brainstem Circuitry Regulating Phasic Activation of Trigeminal Motoneurons during REM Sleep

**DOI:** 10.1371/journal.pone.0008788

**Published:** 2010-01-20

**Authors:** Christelle Anaclet, Nigel P. Pedersen, Patrick M. Fuller, Jun Lu

**Affiliations:** Division of Sleep Medicine, Department of Neurology, Beth Israel Deaconess Medical Center and Harvard Medical School, Boston, Massachusetts, United States of America; Vanderbilt University, United States of America

## Abstract

**Background:**

Rapid eye movement sleep (REMS) is characterized by activation of the cortical and hippocampal electroencephalogram (EEG) and atonia of non-respiratory muscles with superimposed phasic activity or twitching, particularly of cranial muscles such as those of the eye, tongue, face and jaw. While phasic activity is a characteristic feature of REMS, the neural substrates driving this activity remain unresolved. Here we investigated the neural circuits underlying masseter (jaw) phasic activity during REMS. The trigeminal motor nucleus (Mo5), which controls masseter motor function, receives glutamatergic inputs mainly from the parvocellular reticular formation (PCRt), but also from the adjacent paramedian reticular area (PMnR). On the other hand, the Mo5 and PCRt do not receive direct input from the sublaterodorsal (SLD) nucleus, a brainstem region critical for REMS atonia of postural muscles. We hypothesized that the PCRt-PMnR, but not the SLD, regulates masseter phasic activity during REMS.

**Methodology/Principal Findings:**

To test our hypothesis, we measured masseter electromyogram (EMG), neck muscle EMG, electrooculogram (EOG) and EEG in rats with cell-body specific lesions of the SLD, PMnR, and PCRt. Bilateral lesions of the PMnR and rostral PCRt (rPCRt), but not the caudal PCRt or SLD, reduced and eliminated REMS phasic activity of the masseter, respectively. Lesions of the PMnR and rPCRt did not, however, alter the neck EMG or EOG. To determine if rPCRt neurons use glutamate to control masseter phasic movements, we selectively blocked glutamate release by rPCRt neurons using a Cre-lox mouse system. Genetic disruption of glutamate neurotransmission by rPCRt neurons blocked masseter phasic activity during REMS.

**Conclusions/Significance:**

These results indicate that (1) premotor glutamatergic neurons in the medullary rPCRt and PMnR are involved in generating phasic activity in the masseter muscles, but not phasic eye movements, during REMS; and (2) separate brainstem neural circuits control postural and cranial muscle phasic activity during REMS.

## Introduction

Rapid eye movement (REM; also termed paradoxical or active) sleep in the rat is characterized by tonic phenomena including desynchronization of the cortical electroencephalogram (EEG), prominent hippocampal theta activity and postural muscle atonia [1], [2]. In addition, and superimposed on the background of tonic postural muscle atonia during REM sleep, are phasic cranial muscle twitches that include rapid eye movements and twitching of the jaw, whisker and tongue muscles [3]–[6]. Given that the jaw and tongue muscles may share similar neural control mechanism, elucidating the neural mechanisms mediating phasic activation and tonic inhibition of jaw muscles during REM sleep may help to understand the neural control of the tongue muscle in sleep apnea [7]. As a related example, suppression of postural muscle activity during REM sleep prevents sleeping individuals from ‘acting out their dreams’ as occurs in human REM sleep behavior disorder (RBD) [8]. It has been recently shown that damage to the sublaterodorsal nucleus (SLD) in rats and the subcoeruleus region in human [9]–[12] results in the development of RBD. By contrast, the neural circuits mediating both phasic activation and the tonic inhibition of the cranial musculature during REM sleep remain undetermined.

Mo5 is the largest cranial motor nucleus and has two functional divisions: a ventromedial region containing motoneurons innervating the jaw opening muscles and a dorsolateral region containing the motoneurons of jaw closure [13]. The jaw-closing masseter muscles exhibit prominent phasic activity during REM sleep [14], which is blocked by reverse microdialysis of glutamate receptor antagonists into Mo5 [5]. Mo5 receives glutamatergic projections predominantly from the parvocellular reticular nucleus (PCRt) but also a less dense input from the paramedian reticular area (PMnR) [7], [15]. We hypothesized that the PCRt and PMnR are primary sources of glutamatergic input to the Mo5 that drive phasic activity during REM sleep. We tested this hypothesis using two experimental approaches. In the first experiment, we made cell-body specific lesions in the rat SLD, PMnR and PCRt and then recorded the EEG, neck electomyogram (EMG), masseter EMG and electrooculogram (EOG). In the second experiment, we used genetically modified mice to selectively eliminate glutamatergic neurotransmission by rostral PCRt (rPCRt) neurons and subsequently recorded the EEG, neck EMG and masseter EMG.

## Results

### Cell-Body Specific Lesions and Histology in Rats

**Figure 1:**
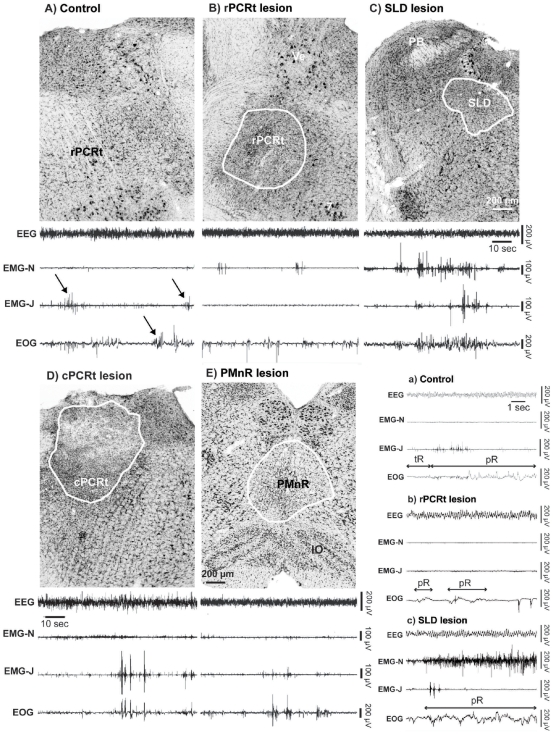
Lesion histology and REM sleep phenotype following cell-body specific lesions in rats. (A) unlesioned control, the arrows show masseter twitches (EMG-J) and bursting in the EOG during REM sleep (B) rPCRt lesion (OX-SAP,0.1%, 330 nl; rPCRt is defined as the portion of PCRt that is rostral to the inferior olive and caudal to the facial tract; cPCRt extends caudally from this), (C) SLD lesion (OX-SAP,0.1%, 130 nl), (D) cPCRt lesion (OX-SAP,0.1%, 330 nl) and (E) PMnR lesion (OX-SAP,0.1%, 230 nl). For each experimental condition a 2 min REM sleep episode example (from the 256 Hz recording) is shown to illustrate the typical EEG, EMG-neck, EMG-jaw and EOG recordings. A set of extended recording examples (from the 256 Hz recording) is showed in the lower right panel and includes: a) control, b) rPCRt lesion and c) SLD lesion cases. Note that during REM sleep, (1) control rats showed activated EEG, EMG-N atonia, EMG-J phasic twitches and rapid eye movements; (2) rPCRt and PMnR lesions eliminated/reduced EMG-J twitches; (3) SLD lesions increased phasic activity of the neck muscles; and (4) cPCRt lesion did not alter the REM sleep EEG/EMGs/EOG. SLD: sublaterodorsal nucleus; PCRt: parvocellular reticular nucleus; PMnR: paramedian reticular area; PB: parabrachial nucleus; Ve: vestibular nucleus; 7: facial nucleus; IO: inferior olive; EEG: electroencephalogram; EMG-N: neck electromyogram; EMG-J: masseter or jaw electromyogram; EOG: electrooculogram; tR: tonic REM sleep; pR: phasic REM sleep.

**Figure 2:**
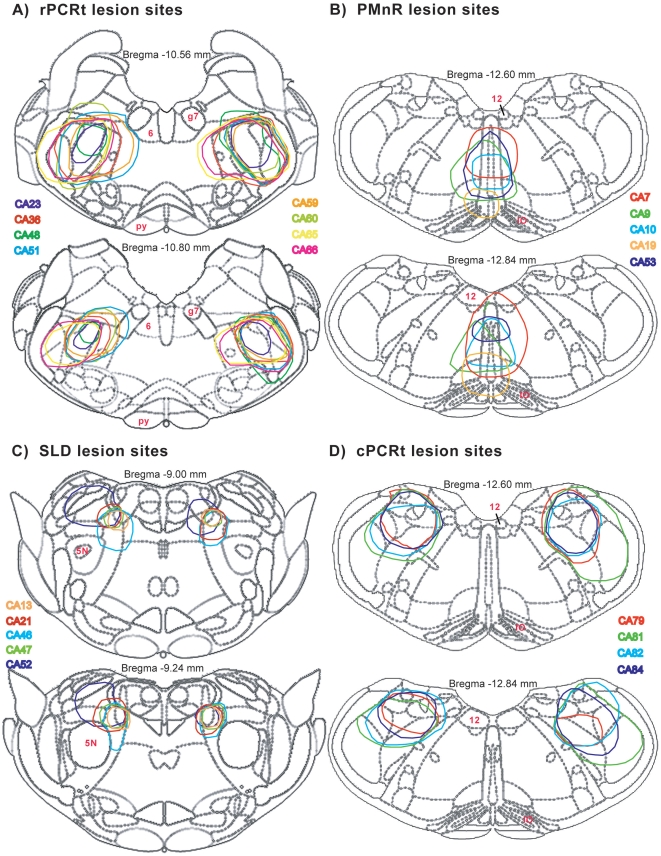
Lesion maps for all rPCRt(A), PMnR(B), SLD(C) and cPCRt (D) cases in rats. 5N: trigeminal nucleus; 6: abducens nucleus; 12: hypoglossal nucleus; g7: facial nerve; IO: inferior olive; Py: pontine nuclei.

We used anti-orexin-B IgG-saporin (OX-SAP) to produce cell-body specific lesions in the rPCRt (rostral to the inferior olive and posterior to the facial nerve) (Fig. 1B), SLD (Fig. 1C), caudal PCRt (cPCRt) (Fig. 1D) and PMnR (Fig. 1E) (see Fig. 2A&D for location of rostral and caudal PCRt). In the PCRt, SLD and PMnR, OX-SAP killed most, if not all, neurons. The extent and boundaries of the lesions were determined by Nissl staining (Fig. 1). All lesion cases for the rPCRt, PMnR, SLD, and cPCRt are illustrated in Figure 2, where we show the overlapping lesions at two levels of each target structure.

### The Effects of Lesion Sites on REM Sleep and Masseter Phasic Activity in Rats

Similar to control rats (n = 13), SLD (n = 5), PMnR (n = 5), rPCRt (n = 8) and cPCRt (n = 4) lesioned rats exhibited a normal diurnal rhythm of REM sleep, with higher levels during the light period. No difference in daily REM sleep time was found between the control and lesion groups (control: 7.5±0.3%, n = 13; SLD: 8.0±0.5%, n = 5, p = 0.4; PMnR: 7.8±1.0%, n = 5, p = 0.8; rPCRt: 6.6±0.4%, n = 8, p = 0.1; cPCRt: 7.1±0.4%, n = 4, p = 0.4; individual t-tests between control and each lesion site). Other motor activities including reflex activities, blinking and head turning appeared normal during wakefulness in all groups.

**Figure 3:**
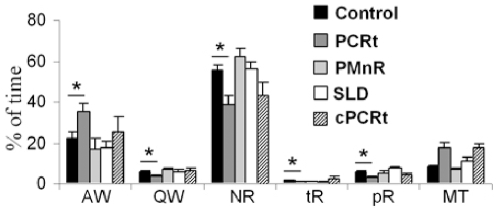
Three hours (9am-12pm) sleep-wake distribution following cell-body specific lesions in rats. Percentage of time of AW: active wake, QW: quiet wake, NR: non REM sleep, tR: tonic REM sleep, pR: phasic REM sleep and MT: artifacts during the 3 h, 2 kHz recording for EMG analysis. All values are means ± SEM; *p<0.05 using a two-tailed t-test.

**Figure 4:**
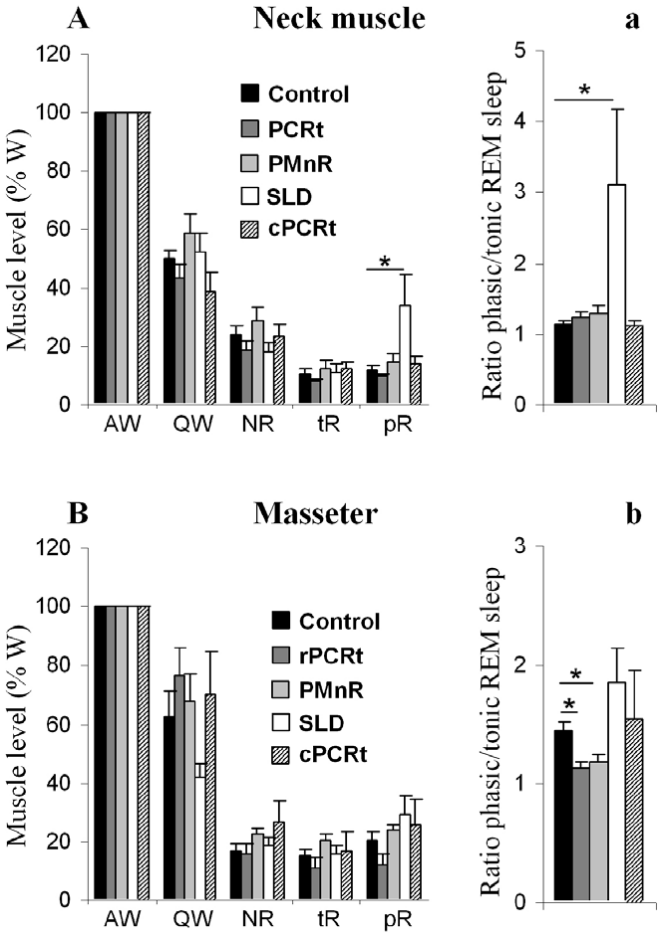
Neck muscle and masseter activity following cell-body specific lesions in rats. In A (neck) and B (jaw), muscle activity for each stage is expressed in percentage of the active wake muscle activity (left panel). a and b (right panel) represent corresponding ratios of phasic and tonic REM sleep muscle activity. AW: active wake; QW: quiet wake; NR: non REM sleep; tR: tonic REM sleep; pR: phasic REM sleep. All values are means ± SEM; *p<0.05 using a two-tailed t-test.

**Figure 5:**
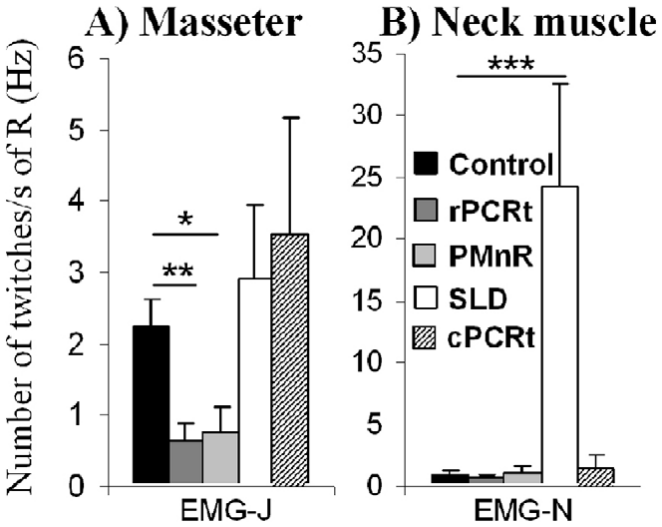
Neck muscle and masseter twitches during REM sleep following lesions in rats. Y axis = number of muscle twitches/REM sleep time (s). Note that SLD lesion significantly increase neck muscle twitches while both PMnR and rPCRt lesions significantly decrease masseter twitches, as compared with the control groups. EMG-J: jaw or masseter electromyogram; EMG-N: neck electromyogram; R: total REM sleep; All values are means ± SEM; *p<0.05; **p<0.01; ***p<0.001 using a two-tailed t-test.

Typical REM sleep from a control animal (Fig. 1A & a) was characterized by a desynchronized EEG with increased power in the theta range, atonia of neck and jaw (masseter) muscles, phasic masseter twitches and rapid eye movements. As others have done previously, we further divided the behavioral state of REM sleep into two components [16]–[18]: tonic REM sleep, which consists of constant atonia of the neck, masseter and ocular muscle (a single muscle twitch may occasionally occur; Fig. 1a); and phasic REM sleep, which is operationally defined as an epoch containing a burst of muscle contractions superimposed on muscle atonia (see Fig. 1A, arrows), and was primarily observed in the masseter and extraocular EMG (Fig. 1A, a). To quantify EMG changes, we recorded jaw and neck EMG at a high sampling rate (2000 Hz) between 9:00 AM and 12:00 PM - a period during which REM sleep is enriched [19], [20]. During this period, only rPCRt lesioned rats showed a significant increase of active wake at the expense of all other stages (Fig. 3). When normalized to active wake, the neck EMG showed a progressive decrease in muscle tone from active wake to tonic REM sleep (Fig. 4A). During phasic REM sleep, the neck muscles showed only a few phasic events (Fig. 1A) (phasic/tonic EMG ratio = 1.15±0.04, n = 10 controls; Fig. 4a). As with postural muscles, masseter muscle tone decreased progressively from active wake to quiet wake and to non-REM (NREM) sleep. However, in contrast to postural muscles, no significant change in tone was seen between NREM and tonic REM sleep (masseter activity as a percentage of quiet wake activity: 17.1±2.1 vs 15.0±2.4% in NREM and REM sleep respectively, n = 10 controls, p = 0.52, Fig. 4B). In addition, during phasic REM sleep, phasic activity in the masseter EMG occurred far more frequently than in the neck EMG (masseter = 3.94±0.65 twitches/s of phasic REM sleep; neck muscle = 1.28±0.43 twitches/s of phasic REM sleep, n = 10 control rats, p = 0.005; Fig. 5).

As reported previously [9], rats with SLD lesions exhibited REM sleep without atonia (i.e., jerking, twitching, jumping and running), the animal equivalent of human RBD. However, we found that the tonic and phasic activity of masseter EMG in the SLD lesioned rats did not differ from that of controls (Fig. 1C). Moreover, the phasic twitches seen in the masseter did not appear synchronized with the neck EMG, nor with the EOG following SLD lesions. PMnR and rPCRt lesions reduced and eliminated masseter phasic twitches, respectively (Fig. 1E & B). Surprisingly, and in contrast to that seen in rPCRt lesioned rats (Fig. 1B), cPCRt lesions had no effect on masseter phasic activity during REM sleep (Fig. 1D). Finally, the rapid eye movements of REM sleep were preserved in all lesion groups, as determined from the EOG (Fig. 1).

In agreement with our observation using the slower 256Hz sampling rate, EMG analysis at 2000 Hz showed that SLD lesions significantly increased neck muscle tone as compared with controls, particularly during phasic REM sleep (Fig. 4A & a). However, SLD lesions did not affect the masseter EMG during REM sleep (Fig. 4B & b). By contrast, lesions of the PMnR and rPCRt did not significantly alter neck muscle EMG tone during REM sleep but significantly reduced the ratio of phasic REM sleep to tonic REM sleep masseter EMG compared with control animals (1.14±0.05 from n = 8 rPCRt lesion group v. 1.45±0.07 from n = 10 control rats, p = 0.0019; 1.19±0.05 from n = 5 PMnR lesion group v. 1.45±0.07 from n = 10 control rats, p = 0.012; Fig. 4b). In addition, rPCRt lesions produced a larger, though not statistically significant, decrease in phasic masseter tone than PMnR lesions (1.14±0.05 in 8 rPCRt lesioned rats vs 1.19±0.05 in 5 PMnR lesioned rats, p = 0.51; Fig. 4b). Additionally, SLD lesions did not affect masseter phasic activity during REM sleep but increased neck muscle phasic twitch frequency (24.16±8.53 twitches/s of total REM sleep in 4 SLD lesioned rats vs 0.98±0.33 twitches/s of total REM sleep in 10 controls, p = 0.0009, Fig. 5). PMnR and rPCRt lesions did not affect neck muscle twitch frequency but significantly decreased masseter twitch frequency (PMnR: 0.77±0.32 twitches/s of total REM sleep, n = 5, p = 0.03 and rPCRt: 0.64±0.25 twitches/s of total REM sleep, n = 8, p = 0.002 vs 2.23±0.38 twitches/s of total REM sleep in control, n = 10, Fig. 5). Consistent with the absence of an effect on neck EMG, cPCRt lesions did not alter masseter tonic and phasic activity during REM sleep (Fig. 4 & 5).

Finally, but for reasons that remain unclear, rPCRt lesions, but not cPCRt lesions, resulted in a ca. 40% loss of body weight. The loss of body weight observed in the rPCRt lesionsed animals could be explained, at least in part, by a decrease in food intake, possibly secondary to changes in masseter motor function. We cannot however make a definitive and quantitative statement to this effect as food intake was not measured in our study.

### Genetic Disruption of Glutamatergic Neurotransmission by rPCRt Neurons in Mice

**Figure 6:**
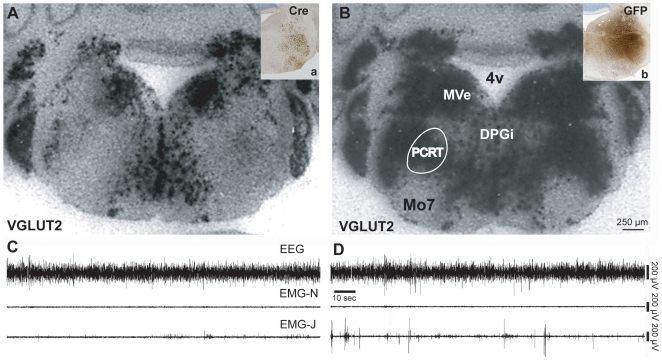
Selective elimination of glutamate release from the rPCRt. Injections (boxes in upper right corners) of AAV-Cre or AAV-GFP encompassing the rPCRt are shown by Cre (**a, knockout**) or GFP (**b, control**) immunostaining in a floxed *Vglut2* mouse. VGLUT2 mRNA signal (black puncta) in an AAV-Cre injected mouse (**A**) and AAV-GFP injected mouse (**B**) in the region corresponding with Cre or GFP immunolabelling in the *Vglut2* mouse. Note the loss of VGLUT2 mRNA in the Cre, but not GFP, injected animal. **C & D**, EEG, EMG-Neck and EMG-Jaw traces during REM sleep in an AAV-Cre injected VGLUT2 floxed mouse (**C**) and AAV-GFP (vector control) injected VGLUT2 floxed mouse (**D**). Specific elimination of *Vglut2* from the rPCRt neurons resulted in reduced phasic masseter twitches during REM sleep.

**Figure 7:**
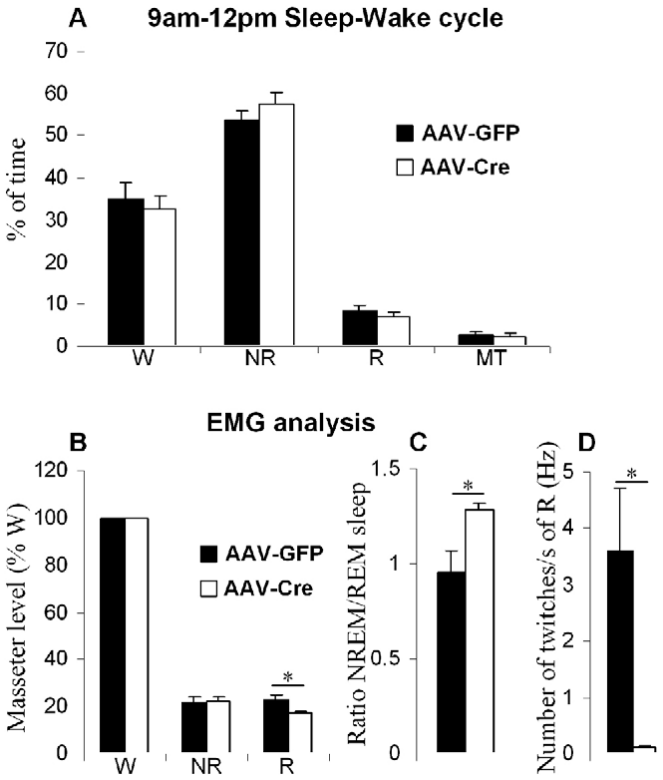
Sleep-wake and masseter activity analysis following loss of rPCRt glutamate release. (**A**) Percentage of W: wake, NR: non REM sleep, R: REM sleep and MT: artifacts from the 2 kHz recording for EMG (3 hours, 9AM-12PM). (**B**) Masseter activity of each stage expressed as a percentage of wake masseter activity in AAV-Cre and AAV-GFP (control) injected mice. (**C**) Ratio of NR and REM sleep masseter activity in AAV-Cre and AAV-GFP injected mice. (**D**) masseter twitches during REM sleep in AAV-Cre and AAV-GFP injected mice. Y axis = number of muscle twitches/REM sleep time (s). All values are means ± SEM; *p<0.05 using a two-tailed t-test.

An adeno-associated viral (AAV) vector containing Cre-recombinase (AAV-Cre) was injected into the rPCRt of transgenic mice with lox-P sites flanking the second axon of the vesicular glutamate transporter 2 gene (VGLUT2-flox). This resulted in a complete loss of VGLUT2 mRNA in the mouse rPCRt (Fig. 6A), as determined by *in situ* hybridization. By contrast, vector control injections of an AAV containing Green Fluorescent Protein (AAV-GFP) did not result in any changes in VGLUT2 mRNA levels (Fig. 6B). Importantly, the regional loss of *in situ* signal overlapped with Cre immunolabeling (Fig. 6a). Analysis of EEG and masseter EMG (EMG-J) recordings showed that selective elimination of glutamate release from rPCRt neurons produced a significant reduction in phasic masseter activity during REM sleep, similar to cell-body specific lesions in the rPCRt (Fig. 6C). VGLUT2 deletion in the rPCRt did not however significantly alter the 3 h (9am-12pm) sleep-wake distribution as compared to control AAV-GFP injected mice (Fig. 7A). The masseter integral EMG (integrated EMG-J) values during REM sleep were decreased in VGLUT2 deleted mice (17.2±0.9% of wake, n = 4, p = 0.016), as compared to controls (23.1±2.0% of wake, n = 3; Fig. 7B). Similarly, the masseter integral ratio of NREM and REM sleep was significantly increased in the VGLUT2 deleted group when compared to the control group (1.28±0.04 vs 0.96±0.11 in control, p = 0.034; Fig. 7C). Finally, VGLUT2 deletion in the rPCRt resulted in the loss of masseter twitches during REM sleep (0.12±0.02 vs 3.59±1.12 Hz in control, p = 0.027; Fig. 7D). Similar to the rPCRt lesionsed rats, body weight was significantly decreased following VGLUT2 deletion (ca. -16%; 21.0±0.7 g, 27±1.2 days after AAV-cre injection, n = 4 v. 25.0±0.6 g, 33±1.5 days after AAV-GFP injection, n = 3; p = 0.009).

## Discussion

Here we report that, in rats, cell-body specific lesions in the rPCRt and PMnR, but not cPCRt, significantly reduced masseter phasic activity during REM sleep without affecting neck muscle activity. By contrast, cell-body specific lesions in the SLD produced jerking and twitching of postural muscles (i.e., REM sleep without atonia; as reported previously, [9]), but did not affect masseter (tonic or phasic) activity during REM sleep. None of the lesions affected daily REM sleep time or rapid eye movements, i.e. EOG activity. AAV-Cre mediated deletion of the VGLUT2 gene in the rPCRt, and hence glutamate release by rPCRt neurons, resulted in the near complete elimination of masseter twitches during REM sleep in mice. These results indicate that, during REM sleep: (1) different neural circuits mediate twitches (phasic) and atonia (tonic) events in the masseter; (2) different neural circuits are involved in masseter (cranial muscle) and neck muscle (postural muscle) twitch control; (3) different neural circuits are involved in generating rapid eye movements and masseter twitches (i.e., two separate cranial muscle control systems); and (4) glutamatergic rPCRt premotoneurons mediate masseter twitches.

### Circuitry Controlling Cranial and Postural Motor Systems during REM Sleep

Parsing muscle function into two separate processes, tonic (atonia) and phasic (muscle twitches), facilitates an understanding of REM sleep muscle control. First, atonia occurs in both postural and cranial motor systems during REM sleep. While the general consensus is that GABA, glycine and monoamines contribute to atonia (i.e., non-phasic) control [21]–[26], the precise role of these neurotransmitter systems has not been resolved, and the neural circuitry responsible for atonia has not yet been elaborated.

As compared to atonia control, the neural basis of phasic control is much better understood. One aspect of phasic control that appears to be conserved between cranial and postural muscles is the role of glutamate in driving phasic activity (the present study and [27]). By contrast, unconserved aspects of cranial and postural phasic control include: 1) the involvement of different glutamatergic sources and neural circuits; and 2) that glycine appears to inhibit phasic activity in postural muscles [21], while glycine is unable to suppress phasic activity in cranial motor systems [28]. For instance, infusion of GABA-A and glycine receptor antagonists into Mo5 increases phasic activity of the masseter during REM sleep [28]. These data suggest that, unlike postural motor systems, Mo5 (and possibly other cranial motor nuclei) may lack active mechanisms for suppressing phasic activity. Postural muscle phasic activity is more completely suppressed during REM sleep, although this varies by age, species and in some neurodegenerative disorders that result in RBD [8]. Our laboratory has previously identified glutamatergic SLD and supraolivary medulla (SOM) [9], [27] neurons that are involved in the phasic control of postural muscles. In the present study we show that the neurons of the rPCRt and PMnR mediate masseter phasic activity during REM sleep, and that this regulation occurs via glutamatergic projections, at least from the rPCRt.

### Medullary Neural Circuitry Regulating Tonic and Phasic Activity in Cranial Muscles during REM Sleep

We have identified two brainstem premotor areas, the PMnR and PCRt, which are involved in generating masseter twitches during REM sleep. Interestingly, cell-body specific lesions of these regions had no effect on masseter atonia, indicating that Mo5 premotoneurons in these regions are not involved in generating masseter atonia during REM sleep. It is therefore likely that separate neural circuits are responsible for producing tonic and phasic phenomena in the masseter EMG during REM sleep. Recently, it was suggested that cholinergic and monoaminergic systems may be involved in generating tonic atonia in cranial motor systems [18], [29], [30]. During REM sleep, Mo5 neurons receive large amplitude inhibitory postsynaptic potentials (IPSPs) [31], most likely mediated by GABA and glycine, which result in masseter atonia, though these conclusions remain controversial [28]. Fenik and colleagues have also proposed an alternative hypothesis in which the loss of monoamines (norepinephrine and serotonin) release is likely responsible for the reduction in tonic muscle tone during REM sleep [23].

While atonia occurs in almost all non-respiratory muscles during REM sleep, phasic activity occurs primarily in cranial muscles and those of the extremities. In the present study, visual observation of the recording traces evidenced no temporal synchronization between phasic masseter and neck phasic EMG activity (SLD lesions) and, furthermore, no temporal synchronization was apparent among neck, jaw and ocular muscles in control rats. By contrast, postural muscle twitching (such as neck and leg) appeared synchronized in SLD lesioned animals (Lu, unpublished data). These results suggest that postural phasic control (excitation and inhibition) originates from a common source (i.e., the SLD) while cranial phasic control likely originates from multiple, discrete sources. While it is not clear what the generator of phasic activity might be for postural muscles, we speculate that it is comprised of local glutamatergic neurons in the spinal cord. Nevertheless, we do know that SLD glutamatergic neurons are involved in preventing the occurrence of phasic movements of postural muscles [32], [33]. Our results suggest that the cell populations driving cranial muscle twitching during REM sleep are probably situated close to the relevant motor nuclei.

The PCRt contains cholinergic, GABAergic/glycinergic and glutamatergic neurons, topographically targeting the hypoglossal, facial and trigeminal motor nuclei [34]. However, the glutamatergic (VGLUT2-positive) population constitutes the largest population of premotor neurons targeting Mo5 [7] and results from the present study demonstrate that glutamate released from PCRt neurons drives Mo5 phasic activity. As the PCRt also contains glutamatergic premotor neurons for facial (Mo7) and hypoglossal nuclei (Mo12) [7], we speculate that glutamatergic neurons in the PCRt may drive phasic activity of Mo7 and Mo12 during REM sleep. Notably, none of the lesions produced changes in phasic EOG activity, suggesting that premotor neurons involved in generating rapid eye movements are not located in the PCRt, PMnR or SLD. Finally, the identities of the inputs that activate the rPCRt/PMnR neurons during REM sleep remain unresolved.

In conclusion, we have identified that glutamatergic neurons of the PCRt-PMnR provide critical inputs to Mo5 for the regulation of phasic masseter activity during REM sleep, although whether or not these putative premotor neurons are sufficient for eliciting or timing this phasic activation remains to be determined. In addition, rPCRt-PMnR putative premotor neurons are not similarly involved in the regulation of atonia or phasic activity of the extraocular and neck muscles, further suggesting that separate neural circuits mediate phasic and tonic atonia of cranial and postural muscles during REM sleep.

## Materials and Methods

### Experiment 1: Lesion Studies in Rats

#### Animals

Pathogen-free, adult, male Sprague-Dawley rats (275–300 grams, Harlan) were individually housed at 20–22°C on a 12:12 light-dark cycle (lights on at 07:00 A.M.), with food and water available ad libitum. The rats were housed in the Harvard Animal Research Facility and the care of animals used in this study met National Institutes of Health standards, as set forth in the Guide for the Care and Use of Laboratory Animals and was approved by the Beth Israel Deaconess Medical Center and Harvard Medical School Institutional Animal Care and Use Committees.

#### Lesions

Because ibotenic acid injection in the medulla produces almost 100% mortality in rats (Lu, unpublished data), in this study, we used anti-orexin-B IgG-saporin (OX-SAP) to produce cell-body specific lesions in different brain areas. OX-SAP is designed to specifically target and kill neurons that express orexin2 (OX2) receptors. Given that OX2 receptors are widely distributed in the brain [35], OX-SAP is capable of producing cell death in most CNS regions. Selectivity is evident at moderate doses (0.05%), with destruction of neurons in the tuberomammillary nucleus and non-cholinergic neurons in the basal forebrain and pontine tegmentum [29]. At higher doses (0.1%), however, OX-SAP may also kill neurons that contain either low levels of OX2 receptors or none at all, such as basal forebrain cholinergic neurons ([27] & Fuller and Lu, unpublished data). However, some regions that lack OX2 receptors, such as the suprachiasmatic nucleus and thalamus are almost completely resistant to the toxic effect of OX-SAP, even at very high concentrations (Lu, unpublished data).

After the animals were anesthetized with chloral hydrate (7% in saline, 350 mg/kg, intra peritoneal), the rats were placed in stereotaxic apparatus, the skull was exposed and a small craniotomy was made. A fine glass pipette [1 mm glass stock, tapering slowly to a 10–20 µm tip] containing anti-orexin-B IgG-saporin (0.1% solution, Advanced Targeting Systems, CA) was then lowered to one of the four predetermined targets, including: sublaterodorsal nucleus [SLD, anteroposterior (AP) −9.24 mm, lateral (L)±1.2 mm, dorsoventral (DV) −6.4 mm, 130 nl], rostral parvocellular reticular nucleus [(rPCRt), AP -10.5 mm, L±2.1 mm, DV -7.2 mm, 330 nl], paramedian reticular area [(PMnR) AP -12.8 mm, L 0.0 mm, DV -8.2 mm, 230 nl] and caudal parvocellular reticular nucleus [(cPCRt), AP -12.3 mm, L±2.0 mm, DV -6.3 mm, 330 nl] as per the rat atlas of Paxinos and Watson (2005; [36]). The orexin-SAP was injected using a compressed air delivery system described previously [37], [38]. In brief, the meniscus within the pipette was directly visualized with an operating microscope, and fluid was ejected using brief puffs of air at 50 lb/ft^2^. These air puffs were regulated by an electronically controlled air switch, which opened for 5–10 ms every 0.5–1 s. These variables were adjusted for each pipette such that 130–330 nl was injected over a 2-min period. The injected volume was determined by directly measuring the movement of the meniscus within the pipette with a reticule on the microscope. After the brain injection, animals were implanted by EEG/EMGs/EOG electrodes.

#### EEG/EMG leads implantation

Four electroencephalogram (EEG) screw electrodes (Plastics One) were implanted into the skull, in the frontal (2) and parietal bones (2) of each side. Two flexible electromyogram (EMG) wire electrodes (Plastics One, stock # E363/76) were placed into the neck muscles via a small needle hole and two additional flexible EMG wire electrodes (Plastics One, stock # E363/76) were placed into the left masseter. For lead placement in the masseter, the muscle was exposed with a small incision in the skin of the left jaw, the head of the electrodes were introduced into the masseter via a small needle hole and the wires were tunneled subcutaneously along the dorsal surface of the cranium. The remaining two flexible EMG wire electrodes (Plastics One, stock # E363/76) were positioned in the medial and lateral canthi of the right eye for recording the electrooculogram (EOG). Each EMG electrode was securely fixed with 0.5 µl of tissue adhesive (LiquiVet, Oasis Medical Mettawa, Illinois). Finally, the free ends of the leads were connected to two pedestal sockets (6 pin connector, Plastics One) and the assembly was fixed in place with dental cement. The scalp wound was closed with surgical clips, and the rat was kept in a warm environment until resuming normal activity. The entire procedure was completed in 60–90 min.

#### Sleep-wake recording

One week after surgery, the sockets were connected via flexible recording cables and a commutator (Plastics One) to an analogue amplifier (A-M Systems) and computer, with an analogue to digital converter card and running VitalRecorder (Kissei, Japan). EEG/EMGs/EOG was recorded at the end of the second post-surgical week for two separate 36 h recording sessions, beginning at 7:00 P.M. and at a sampling rate of 256 Hz (daily sleep-wake cycle analysis) and for two separate 3 h recording sessions, beginning at 9:00 A.M. and at a sampling rate of 2000 Hz (EMG analysis). No filter was applied for any recording. During each recording session, the animals were left undisturbed.

#### Perfusion and histology

At the end of each experiment, animals were deeply anesthetized by chloral hydrate (7% in saline, 500 mg/kg, intra peritoneal), and perfused though the heart with 50 ml saline followed by 500 ml 10% formalin in phosphate buffer saline (PBS). The brains were removed, post-fixed in 10% formalin in PBS overnight, equilibrated in PBS containing sodium azide (0.02%) (PBS-Azide) and sucrose (20%) for 3 days, and then sectioned at 40µm on a freezing microtome in four series. Brain sections were mounted, dried, stained with thionin, dehydrated and coverslipped. Tissue sections were viewed using a light microscope and the location of the lesions was plotted on standardized brain maps [30] to verify the lesion localizations.

#### Control group

Six rats without any brain injection were implanted with EEG/EMG-neck/EMG-masseter/EOG to study normal REM sleep muscle phenotypes. Four rPCRt and three SLD OX-SAP injected rats failed to display any REM sleep phenotype and subsequent Nissl staining confirmed that these animals also did not have lesions in these or other areas. Because the results from both non-injected and non-lesioned rats were not statistically different, they were pooled together and are reported as one control group. In addition, three rats within the control group had considerable heart muscle activity artifacts in the EMGs recordings. Therefore these rats were include in the sleep-wake time analysis (n = 13 controls) but were excluded from the EMG analysis (n = 10 controls).

#### Behavioral states

Three behavioral states (Wake, NREM and REM sleep) were visually identified and analyzed in 10-s epochs for daily sleep-wake cycle analysis (sampling rate: 256 Hz); six behavioral states (AW, QW, NR, tR, pR and MT; see below) were visually identified and analyzed in 4-s epochs for the EMG analysis (sampling rate: 2000 Hz) using SleepSign for Animal (Kissei, Japan). Classification of behavioral states was as follows: active wake (AW), characterized by high frequency, low-voltage EEG signals coupled with high levels of EMG activity (i.e., chewing, grooming, drinking); quiet wake (QW), characterized by high-frequency, low-voltage EEG signals and an absence of overt motor activity or a change in sleeping position coupled with a minimum of 4-s EEG activation; NREM sleep (NR) was characterized by high-amplitude, low-frequency EEG signals and the absence of motor activity; tonic REM sleep (tR) was characterized by low-amplitude, high-frequency theta-like EEG activity and neck and jaw muscle atonia, when muscle twitches and rapid eye movements are conspicuously absent, episodes included isolated muscle twitch and/or isolated eye movement where scored as tR; phasic REM sleep (pR) was characterized by low-amplitude, high-frequency theta-like EEG activity and neck and jaw muscle atonia interspersed by periodic burst of muscle twitches and/or burst of rapid eyes movements during more then 50% of the 4-s epoch. State transitions, sleep jumps or positional changes associated with an activated EEG shorter than 4-s were removed from the analysis and scored as artifact (MT).

#### EMG analysis

REM sleep consists of both tonic and phasic motor events. Tonic REM sleep constitutes the stereotypical periods of motor atonia during REM sleep while phasic REM sleep constitutes the periodic muscle twitches that punctuate REM sleep (i.e., during rapid eye movements) [3], [6]. Because a major goal of this study was to determine the importance of SLD, PMnR and/or PCRt projections in modulating motor activity in REM sleep, we developed an objective method for quantifying the tonic (i.e., REM sleep atonia) and phasic (i.e., muscle twitches) periods of REM sleep. To quantify muscle tone, raw EMG signals were filtered between 0–10 Hz in SleepSign for Animal using a high pass filter, which eliminated body movement artifacts, and were full-wave rectified, integrated, and quantified in arbitrary units, for every 4-s epoch, using the “integral” function of SleepSign for Animal. Then all measurements were normalized as mean activity during all of AW being 100% (Fig. 4A&B) or as mean activity during all of tR being 1 (Fig. 4a&b). The muscle twitches that define phasic REM sleep were detected as motor events that exceeded at least two times the amplitude of EMG activity during tonic REM sleep. In both lesioned and control rats, muscle twitches were quantified by 4-s epochs for each REM sleep epochs, using the “cross level” function of SleepSign for Animal.

### Experiment 2: Genetic Deletion of the Vesicular Glutamate Transporter 2 (VGLUT2) Gene from the rPCRt in Mice

#### Microinjection and recordings

Experiment 2 was performed using mice with loxP sites flanking exon-2 of the Vglut2 gene (n = 7). To selectively eliminate glutamate neurotransmission by rPCRt neurons in these mice, we injected an adeno-associated viral (AAV) vector expressing Cre recombinase (Cre) into the rPCRt (AP = −5.5, DV = −4.5, RL = ±1.3 mm; [39]) (n = 4). When exposed to Cre, the second exon of the VGLUT2 gene was deleted, rendering the Cre-infected neurons incapable of expressing functional protein and hence preventing vesicular neurotransmitter (glutamate) packaging and subsequent release of glutamate from rPCRt neurons [40]. As vector injection controls, we injected AAV expressing green fluorescent protein (GFP) into the rPCRt of Vglut2 mice (n = 3). Injections of AAV-Cre or AAV-GFP into the rPCRt of these mice were done using a compressed air delivery system [37], [41]. After injections of either AAV-Cre (60 nL) or AAV-GFP (60 nL), mice were implanted by EEG/EMG-neck/EMG-masseter electrodes. Three EEG screw electrodes (Pinnacle Technology Inc., Kansas) were implanted into the skull, in the parietal (n = 1) and frontal (n = 2) bones of each side. One flexible EMG wire electrode (Plastics One) was placed between the neck muscles and two additional flexible EMG wire electrodes (home-made with Cooner Wire, California; head diameter = 1 mm) were placed into the left masseter. For implanting the masseter EMG, a small incision was made in the skin of the left jaw, the head of the electrodes were introduced into the masseter via a small needle hole, the leads were securely fixed with 0.2 µl of tissue adhesive (LiquiVet, Oasis Medical Mettawa, Illinois) and the wires were tunneled subcutaneously along the dorsal surface of the cranium. The free ends of the leads were connected to a connector (Pinnacle Technology Inc.) and the assembly was fixed in place with dental cement. Three weeks after surgery, the connector was connected via a flexible recording cable, preamplifier and commutator to an analogue amplifier (Pinnacle Technology Inc.) and computer. Following a one-week habituation period to the recording box and cabling, EEG/EMGs were recorded for two separate 24 h recording sessions, beginning at 7:00 P.M. at a sampling rate of 400 Hz and two separate 3 h recording sessions, beginning at 9:00 A.M. at a sampling rate of 2000 Hz. During each recording session, the animals were left undisturbed. In brief, the EEG was recorded contralaterally in the fronto-parietal cortex, EMG-Jaw was recorded using the EMG channel of Sirenia (Pinnacle Technology Inc.), EMG-Neck was recorded between the parietal EEG electrode and the neck EMG electrode and was used to assist in the scoring of the data, although the EMG signal was not included in the analysis.

Analyses of mouse sleep–wakefulness and quantification of EMG were similar to that used for the rat recordings, including the use of SleepSign for Animal.

#### In situ hybridization and immunohistochemistry

On completion of the recordings, mice were deeply anesthetized with chloral hydrate and perfused transcardially with saline (50 ml) followed by 10% formalin in phosphate buffer saline (PBS) (200 ml). The brains were removed, post-fixed in 10% formalin in PBS overnight, equilibrated in PBS containing sodium azide (0.02%) (PBS-Azide) and sucrose (20%) for 3 days, and then sectioned at 40 um on a freezing microtome in two series. One series from each brain was processed for VGLUT2 in situ hybridization and the other one for Cre-immunohistochemistry [42]. One series of sections from the animals that received AAV-GFP were processed for GFP-immunohistochemistry as described previously [9], instead of Cre-immunohistochemistry. For the *in situ* hybridization, sections were acetylated and hybridized overnight (55°C) with a ^35^S-labeled cRNA probe synthesized from a plasmid containing the complete coding sequence of VGAT or VGLUT2. After a succession of one hour washes (2xSSC/1 mM DTT, 50°C; 0.2xSSC/1 mMDTT, 55°C; 0.2xSSC/1 mM DTT, 60°C), the tissue was treated with RNase-A and washed under conditions of increasing stringency, including a 30 min wash at 60°C in 0.1xSSC. The tissue was then dehydrated in alcohols and air-dried. The sections were exposed to X-ray film (Eastman-Kodak) for 2–3 days, and then the slides were dipped in Kodak NTB-2 emulsion and exposed for one month. Slides were developed in Kodak D-19, fixed, and then dehydrated and coverslipped. For **Cre- or GFP-immunohistochemistry**, sections were washed in phosphate buffer saline containing triton (0.25%) and sodium azide (0.02%) (PBST-Azide) and then incubated in primary antiserum (Cre antibody, Novagen or GFP antibody, Invitrogen, both at 1∶20,000 in PBST-Azide) for two days at room temperature. Sections were then washed in PBST and incubated in biotinylated secondary antiserum (against rabbit IgG, 1∶1000 in PBST, Vector) for two hours, washed in PBST and incubated in ABC regents for two hours. Sections were then washed again and incubated in solution of 0.06% 3,3-diaminobenzidine tetrahydrochloride (DAB, Sigma) and 0.02% H_2_O_2_.

### Experiment 1 and 2

#### Statistical methods

Statistical differences in sleep-wake time and muscle tone or twitches were assessed using a Student's two-tailed t-test, after a normal distribution was confirmed for each group and a test for variance homoscedasticity was performed. p<0.05 was considered significant.
